# Visual Autism

**DOI:** 10.3390/children10040606

**Published:** 2023-03-23

**Authors:** Margaret Reynolds, Susan M. Culican

**Affiliations:** 1 Department of Ophthalmology and Visual Sciences, Washington University Saint Louis, 660 S. Euclid Ave, St. Louis, MO 63110, USA; 2 Department of Ophthalmology and Visual Neurosciences, University of Minnesota, Minneapolis, MN 55455, USA

**Keywords:** blindism, visual autism, congenital blindness, autism spectrum disorder

## Abstract

Autism spectrum disorder (ASD) is a lifelong neurodevelopmental disorder characterized by deficits in social communication and restricted, repetitive behaviors. It affects approximately 2.2% of children. Both genetic and environmental risk factors have been identified for ASD. Visual comorbidities are relatively common among children with ASD. Between 20 and 44% of ASD children have visually significant refractive error, on-third have strabismus, and one-fifth have amblyopia. In addition, ASD is 30 times more common in children with congenital blindness. It is unknown whether the association of ASD with visual morbidity is causal, comorbid, or contributing. Structural and functional abnormalities have been identified in MRIs of ASD children, and ASD children have been noted to have aberrant eye tracking. ASD children with visually significant refractive errors and poor spectacle compliance (present in 30% of ASD children) offer the opportunity for investigation into how improved visual acuity influences ASD behaviors. In this review, we focus on what is known of the visual system, refractive surgery, and ASD.

## 1. Introduction

Autism spectrum disorder (ASD) is a neurobehavioral disorder associated with restrictive and repetitive behaviors [1,2]. Children with congenital visual impairment (VI) or decreased visual acuity (VA) have long been reported to have an increased risk of ASD. The reason for this increased risk is controversial. It is unknown whether VI is causative or correlative with ASD. Both hypotheses are prevalent in the literature. In this review, we discuss ASD and its relationship with visual impairment, highlighting literature regarding the role of VI in ASD and known structural and functional changes in the visual system associated with ASD. Finally, we highlight the utility of refractive surgery among the ASD population and how refractive surgery may offer insights into the role of ASD in VI.

## 2. Relevant Sections

### 2.1. Cause of Autism Spectrum Disorder (ASD)

Autism spectrum disorder, which affects 2% of individuals in the population, is characterized by deficits in social communication and restrictive and repetitive behaviors [1,2]. Both genetic and environmental risk factors have been identified in individuals with ASD; however, a definitive etiology is unknown. In a population-based study including 22,156 individuals with ASD from 3 Nordic countries, genetics were attributed to 81% of ASD traits, and environmental factors were associated with 14–22% of ASD risk [3]. Variants in 100 genes have been associated with significant ASD risk, while thousands of common variants have been associated with a lesser risk [3]. It is hypothesized that environmental risk factors, in combination with genetic risk factors, play an important role. Environmental risk factors that have been identified include older maternal and paternal age and medication use in pregnancy such as valproic acid [4,5,6]. Another environmental risk factor, sensorial deprivation, has been hypothesized to aggravate ASD [7,8,9]. ASD phenotypes are prevalent among children neglected in orphanages [10].

### 2.2. Screening/Diagnosis

The American Academy of Pediatrics recommends that children aged 18 to 24 months be screened for ASD [11]. Screening tools often used in the primary care setting include the Modified Checklist for Autism in Toddlers, Revised (M-CHAT-R), which is a 20-item screening questionnaire. Children with positive screenings can be referred for evaluation. Diagnosis of ASD is made by experts based on the clinical consensus published in the DSM-V. The Autism Diagnostic Observation Schedule (ADOS-2) is a gold standard for diagnosis of ASD [12,13].

Once diagnosis is made, treatment can be implemented. While no specific pharmacologic treatment is recommended, behavioral interventions are well supported by the evidence [14]. Behavioral therapy focuses on teaching skills in social communication and decreasing repetitive and restrictive behaviors. Earlier interventions are thought to lead to greater response [12,13]. Finally, pharmacologic interventions can mitigate comorbid psychiatric conditions such as behavioral and emotional dysregulation or ADHD [15].

Of note, ASD can be difficult to diagnose in children with decreased visual acuity. A modified version of the ADOS has been published for children with decreased visual acuity [16].

### 2.3. Ocular Comorbidities

Up to 13–71% of children with ASD have been found to have ocular comorbidities, including significant refractive error (42%), strabismus (22–57%), amblyopia (19–31%), optic neuropathy (4%), and nystagmus (3%) [17,18,19,20,21,22]. Population-based studies have found that children with ASD are significantly more likely to have visual morbidity than typical peers [17].

Children who have irreversible blindness are at a higher risk of ASD diagnosis (30×) than sighted children [23]. This association has been long reported. In 1956, Keeler et al. noted that 5/60 (8.3%) premature children with ROP and total blindness had autism [24,25]. Many of the other children with total blindness in this cohort had autistic traits. Since that time, ASD has been reported in association with retinopathy of prematurity (ROP), Leber’s amaurosis (LCA), septo-optic nerve dysplasia, micro-ophthalmia, anophthalmia, and CHARGE syndrome [24,25]. In a prospective study describing children born over the course of a decade in Sweden, ASD was one of the most common additional impairments among children with early onset blindness, and the prevalence differed with diagnosis in children with optic nerve hypoplasia (70%), in children with ROP (58%), in children with microphthalmia/anophthalmia (44%), and in children with LCA (36%) [26].

Whether congenital blindness is causal or correlative is unknown. An argument that congenital blindness is correlative is that some forms of congenital blindness carry a higher risk of ASD, indicating that decreased visual acuity may not be the cause of the risk for ASD but that decreased vision and the ASD phenotype may share a common cause, e.g., genetic risk. In a population-based study that compared the incidence of blindness among children blind secondary to ROP versus children blind due to hereditary retinal dystrophy, those with ROP (14/27) were significantly more likely than those with retinal dystrophy (2/14) to be diagnosed with ASD, potentially implicating prematurity/brain injury rather than decreased visual acuity per se as the primary contributing factor [27]. Similarly, children with optic nerve hypoplasia (ONH) were at increased risk of ASD, irrespective of their visual acuity, leading investigators to hypothesize that the underlying cause of ONH rather than visual acuity resulted in increased risk for ASD [28,29].

Fazzi et al. found that among 214 children with cerebral causes of visual impairment and 59 children with peripheral visual impairment, ASD was more prevalent than in the general population; however, prevalence among the central visually impaired (2.8%) was different than among those with peripheral impairment (8.4%). However, the authors argued that the presence of autistic symptoms was consistent with the diagnosis of ASD only in the subjects with cerebral visual impairment. Children with peripheral visual impairment had overlapping symptoms with visual loss, making a clear diagnosis difficult [30].

Others argue that the cause of decreased visual acuity is irrelevant and that the behaviors shown by children with poor visual acuity are secondary to visual deprivation, causing abnormal visual experience and leading to poor development of self-image and self-representation. In other words, characteristics/ASD traits, while common among children with impaired visual acuity, are a phenocopy of ASD rather than ASD itself. Proponents of this idea propose that the ASD behaviors exhibited by congenitally blind children are the result of the disruption of other “pathways” than those with ASD. In fact, ASD diagnosis criteria can differ for children who are sighted and visually impaired [31]. Children with congenital blindness have impaired sensory experience, which has been hypothesized to lead to reorganization of brain connectivity [23]. Specifically, sensory impairment secondary to congenital blindness could lead to impairment in communication, language, and social development, which could manifest as symptoms of ASD [32,33,34,35,36,37].

Children with ASD and congenital blindness share impairment in social interaction and language skills [38,39,40,41]. Children with decreased visual acuity demonstrate difficulties in peer social interactions, restricted play, echolalic speech, and stereotyped behaviors, which are common to ASD [39,42,43]. These behaviors among blind children are termed “blindisms” because they can be attributed to decreased visual acuity [31]. In a prospective assessment by Goodman and Minne [44] including 17 congenitally blind children (aged 4 to 11 years) without any additional impairment, the prevalence of ASD according to the Autism Behavior Checklist was 23.5%. Brown et al. found a prevalence of 20.8% among 24 congenitally blind children without any “neurological damage” (aged 3 to 9 years) using the CARS [45]. Finally, among age- and verbal IQ-matched children, those who were congenitally blind had similar features to matched autistic children [38].

### 2.4. Joint Attention

A leading abnormality among ASD children that also occurs in blind children is altered “joint attention”. Babies and young children use eye contact, gaze following, and joint attention for communication and to learn behavior and intentions of others, especially during the prelinguistic stage [46]. These behaviors are key for emotional attachments and language [46]. Vision and visual perception are crucial for joint attention [47]. The development of joint attention in visually impaired children is not completely understood, and it is difficult to study, as no standardized measure of joint attention for visually impaired children exists. However, an exploratory study suggests that joint attention develops later and differently in visually impaired children than sighted children [48], and this is considered by some authors to be a sign of ASD [49]. However, ASD may lead to the disruption of joint attention [50].

### 2.5. Language and Communication Skills

Visual acuity is important for early language development, as joint attention driven by vision is thought to provide the framework within which language learning occurs [51]. Visually impaired children have language and communication disorders secondary to visual deficits and difficulties with interactive experiences or communication/language resulting from other neurodevelopmental conditions [30,51,52].

Both VI and ASD children can have difficulty communicating [51]. Similarities in their communication techniques include echolalia and speaking without eye contact.

### 2.6. Eye Tracking/Visual Behaviors

Visual acuity is not the only aspect of the visual system that has been associated with ASD. Eye tracking/eye contact and structural and functional brain changes in the visual pathway have been identified in MRI imaging among ASD children. Grossly, eye movements have been identified as abnormal among ASD cohorts. Among a series of patients, eye tracking assessments were performed and graded as “good” if “fixation tracking and further fixation was good”, as average if “the fixation and tracking was happening, but further fixation was creating a problem”, and as “poor” if either fixation or eye tracking was affected. In one group, pursuit movements were poor in 35 (29.2%), average in 39 (32.5%), and good in 46 (38.3%) students. Saccadic eye movements were good in 100 (83.3%), average in 15 (12.5%), and poor in 5 (4.2%) students [53].

It is possible that difficulty with eye movements contributes to abnormal visual behaviors that have been associated with ASD, such as decreased fixation on the faces and eyes of others. Some two-year-olds with ASD were found to exhibit preferential attention to mouths rather than eyes [54]. This identification led to prospective work that found that 2-month-olds who were later diagnosed with ASD initially exhibited increased eye looking but that eye looking subsequently declined to below the levels of typically developing peers [55]. The hypothesis is that other environmental features such as non-social, physical cues occupy the attention of these infants rather than social, facial cues [56]. Whether this is related to a developmental problem with the visual system or whether visual impairment worsens/contributes to this pathology is unknown.

To explore the mechanisms that cause reduced fixation on eyes among ASD children and typically developing children, Pruett et al. compared results of visual search experience in ASD children versus typically developing children. They found intact basic search mechanisms in ASD children [57]. Others have found that visual search experience is enhanced in ASD children [58]. In spite of intact/enhanced basic search mechanisms, children with ASD had reduced accuracy for eye region search. This finding suggests that eyes contribute less to high-level face representation in ASD or that these children have a disruption in attention to the eye region [57].

### 2.7. Abnormal Visual Behavior in ASD Children with Perfect Visual Acuity

We recognize that children with ASD have abnormal visual behavior even when their visual acuity is 20/20 in both eyes. The exact etiology of abnormal visual behavior is unknown, but it has been widely studied. ASD children are known to exhibit (1) abnormal face recognition and difficulty with composite faces and facial inversion, processing part of the face as opposed to the whole face; and (2) eye avoidance, possibly avoiding looking at eyes [59].

The influence of decreased visual acuity on these behaviors is unknown. It is known that interventions including ABA therapy, training protocols, and computer programming can be performed to improve these behaviors [59].

### 2.8. MRI Findings

#### 2.8.1. Structural

The brain basis for abnormal visual behaviors is unknown. However, structural and functional cortical changes in the visual pathway have been identified in MRIs among ASD children.

In a prospective neuroimaging study of 106 infants at high familial risk of ASD vs. low-risk infants, those with ASD exhibited hyperexpansion of the cortical surface area between 6 and 12 months of age, which preceded brain volume overgrowth at 12–24 months of age. The degree of volume overgrowth was correlated with the severity of ASD traits. The overgrowth of the occipital gyrus was highly predictive of ASD development. These findings demonstrate that early brain changes occur during the period in which autistic behaviors first emerge [60]. In addition, splenium microstructure at 6 months of age predicts autism diagnosis at 24 months and has been implicated in the development of visual orienting [61].

Finally, Girault et al. found that greater levels of proband ASD traits were associated with reduced white matter integrity in components of the visual system in siblings who developed ASD [62]. In addition to structural MRI changes, functional MRI changes in visual circuitry have been identified in ASD children.

#### 2.8.2. Functional MRI

In correlation with reduced white matter integrity, Girault et al. found weaker functional connectivity between several networks and the visual system among all high-risk siblings during infancy who later developed ASD [62]. Multimodal anatomical and functional convergence on networks involved in visual processing suggest that inherited liability has a role in shaping the prodromal development of visual circuitry in ASD [62].

Impaired eye region search accuracy has been identified in children with ASD. Initiating joint attention (IJA) is behavioral engagement of two people. It is important for social communication and related to the development of language, empathy, and theory of mind. Deficits in IJA are associated with ASD. The functional organization of the brain is intimately related to the emergence of IJA. The strongest brain–behavior associations cluster within connections between the visual network and dorsal attention network and between the visual network and posterior cingulate aspects of the default mode network. The implication of these findings is that functional brain systems underlie social behavior and that the cortical visual pathway is important for the development of social behavior and interactions [63]. This association was further supported by functional connectivity imaging, which described an association between ASD behaviors and medial visual networks [64].

### 2.9. Refractive Error and Refractive Surgery in Children

Among children with refractive error and ASD, 31% vs. 4% of typically developing children refuse to wear glasses, respectively [65]. Another study reported that only 19% of ASD children complied with prescribed glasses [53]. Without correction, severe ametropia can cause visual impairment to the level of legal blindness (20/200 or worse). This visual impairment may further hinder the development and quality of life by impairing socialization, motor skills, and interaction with the environment.

Refractive surgery can improve visual acuity in this population and is being performed at a small number of centers throughout the United States [66,67,68]. The procedure is more complex in children than adults because of the ongoing ocular growth of the pediatric eye and compliance with preoperative workup and postoperative care [69]. Pediatric refractive surgery is typically reserved for cases in which traditional treatments for refractive error have failed and has been used to treat anisometropia, bilateral high ametropia, and accommodative strabismus [69].

### 2.10. Refractive Surgery in Children Improves Visual Acuity

Overall, refractive surgery has been shown to be safe in children and to result in improvement of refractive error and improved visual acuity [70].

Corneal surface procedures, excimer laser ablation, and intraocular lens implantation have been used. Surface procedures can be used to treat anisometropia [67,71], hyperopia [72,73], myopia [73,74,75], and astigmatism [72]. All have all been reported to be safe [69]. Three-quarters of eyes have been reported to remain within 3 diopters of their target power after 5 years [74]. Of note, longer-term follow-up would be beneficial, as refractive error can continue to change through the early 20s. However, the goal of any refractive surgery is to prevent irreversible amblyopia. We recognize that children may require additional glasses or procedures in the future; therefore, the risks of surgery versus benefits for amblyopia therapy must be considered. There are risks associated with any procedure, and refractive surgery is no exception. The risk of each procedure differs, e.g., LASIK is associated with increased risk of flap dislocation; however, many centers recommend PRK, which avoids the risk of the flap. Nevertheless, regression, infection, and corneal clouding are known risks of corneal surface ablation [69].

Unfortunately, there are refractive errors that are beyond the limits of excimer laser procedures. For these procedures, intraocular lens implantation can be used. Anisometropic amblyopia [76,77,78], myopia [79,80,81], and hyperopia [68] have been treated with phakic intraocular lenses (pIOL). PIOL placement is reversible. Still, PIOL risks exist, similar to those of any intraocular surgery, including infection, residual refractive error, and increased risk of retinal detachment. Such risks should be weighed against the benefits of the surgery; the degree of amblyopia and current visual functioning should be taken into consideration [78]. Finally, there are refractive errors beyond the limits of pIOL, or children whose anterior chamber depth is too small for pIOL. These children are candidates for clear lens exchange [82]. Clear lens exchange is associated with similar risks as PIOL, including retinal detachment and infection, along with the additional risk of loss of accommodation [83].

### 2.11. Refractive Surgery in Children Improves Quality of Life and Behavioral Outcomes

In addition to improving visual acuity, refractive surgery can improve behavioral and developmental outcomes and quality of life. A recent study by Paysse et al. showed that PRK improves the developmental outcomes of children with intellectual disability and high isoametropia [82]. Similarly, in studies of refractive surgeries in children with neurobehavioral disorders, 85–92% exhibited enhanced visual awareness, attentiveness, or social interactions postoperatively [81,82,83,84]. We recently published the results of a small pilot study enrolling 24 children pre- and 1-month post refractive surgery. All patients underwent surgery without complication and improvement in visual acuity. Quality of life scores as measured by the Pediatric Eye Questionnaire improved by a median of 22 points on the functional vision scale. Additionally, Social Responsiveness Scale-2 (SRS-2) scores improved by a median of 15 points 1 month post refractive surgery. SRS-2 identifies the social impairment within the autism spectrum and differentiates it from other disorders. Improvement on this scale indicates improvement in social deficits secondary to ASD post refractive surgery.

The implications of improved visual acuity for ASD children are unknown to date. However, further understanding of how the ASD phenotype changes secondary to improved visual acuity may offer some insight into how decreased visual acuity affects ASD children. Further investigation is certainly merited. Because uncorrected refractive error is a potentially reversible cause of blindness in children with ASD traits, understanding the behavioral impact of reversing visual impairment in this group will help elucidate the underlying neural mechanisms for this association between ASD and visual impairment.

## 3. Discussion

In summary, ASD is a relatively common neurobehavioral disorder with a prevalence of 2% in the population. Children with ASD have a relatively high rate of ocular comorbidities including refractive error; however, a high percentage (1/3) children refuse to wear their spectacles even though they are rendered legally blind. Refractive surgery in this population has been proven safe and effective in correcting refractive error and improving visual acuity. It has also been shown to improve quality of life, acquisition of milestones, and social responsiveness.

## 4. Conclusions

In conclusion, visual acuity correction via refractive surgery in spectacle-non-compliant ASD children appears to have benefits for visual acuity, as well as for behavior and quality of life.

## 5. Future Directions

The role of visual acuity improvement as a way to improve ASD behaviors is worthy of additional exploration. Additional research should be conducted to determine which patients experience improved quality of life and behavioral benefits as a result of this treatment. Additional research should be conducted on why these individuals benefit from such interventions.

## Data Availability

Not applicable.
